# The quality evaluation of 30 *Asparagus officinalis* L. varieties

**DOI:** 10.1002/fsn3.3971

**Published:** 2024-01-23

**Authors:** Rui Gao, Guanghui Li, Pingxiang Liu, Lei Gao, Jingxiu Bi, Yuying Jiang, Honglei Liu, Yutao Wang

**Affiliations:** ^1^ Shandong Provincial Key Laboratory of Test Technology on Food Quality and Safety, Institute of Quality Standard and Testing Technology for Agro‐Products Shandong Academy of Agricultural Sciences Jinan Shandong China; ^2^ Shandong Provincial Key Laboratory of Crop Genetic Improvement, Ecology and Physiology, Biotechnology Research Center Shandong Academy of Agricultural Sciences Jinan Shandong China; ^3^ State Key Laboratory of Microbial Technology Shandong University Qingdao Shandong China; ^4^ Cooperative of Vegetable and Grain Cultivation Liaocheng Yifeng Bloc Liaocheng Shandong China

**Keywords:** *Asparagus officinalis* L., comprehensive evaluation, flavonoids, free amino acids, protodioscin, rutin

## Abstract

Asparagus, a vital economic contributor, is a well‐liked vegetable grown around the globe, and some secondary metabolites in its spear are beneficial to human health. Asparagus spears possess a significant quantity of nutrients and phytochemicals; however, the difference in these chemical compositions among various varieties has not been sufficiently studied. This work aimed to detect the chemical compositions of 30 varieties of asparagus and to assess them by principal component analysis (PCA). The results showed that the contents of these chemical compositions varied in varieties. Selenium (Se, 1.12–2.9 μg/100 g dry‐weight [DW]) was abundant in asparagus, with an average dry matter content of 8.25%. Free amino acids (5.60–9.98 g/100 g DW) and polyphenols (6.34–8.67 mg/g DW) were both present in high amounts, along with flavonoids (4.218–8.22 mg/g DW) and protodioscin (0.44–1.96 mg/g DW). Correlation analysis, PCA, and hierarchical cluster analysis were used to conduct a comprehensive evaluation of asparagus. Atlas, Appolo, Jinggang 111, Jingke 2, and WS‐1 were the top five varieties with comprehensive scores. This study provided valuable data for the breeding, quality improvement, processing, and utilization of asparagus varieties in the future.

## Graphical abstract

A comprehensive evaluation of 30 asparagus varieties in Shandong Province China was conducted. Asparagus show differences in components depending on their varieties, and their quality varies between stem colors. The top five varieties with comprehensive scores were Atlas, Appolo, Jinggang 111, Jingke 2, and WS‐1.

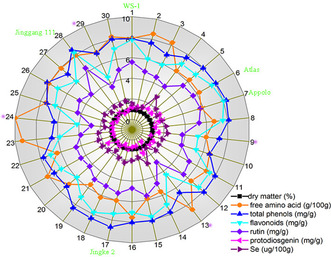

## 1 INTRODUCTION

Asparagus (*Asparagus officinalis* L.), a perennial root herb of the genus Asparagus, is also known as garden asparagus. As an economically valuable plant (Li & Lin, 2022), asparagus has been cultivated and consumed worldwide for a long time. To date, China is the country that cultivates the largest area of asparagus and produces the highest total output in the world. According to Food and Agriculture Organization of the United Nations (2023) statistics, in 2021, the asparagus cultivation area in China was 1,441,009 ha (approximately 91% of the world's total area) and the total production reached 7,344,390 tons (approximately 88% of the global total production). Shandong Province is the place where the majority of Chinese asparagus is produced and exported (Liu et al., 2022).

The main edible part of asparagus is the spear, which is delicious and rich in nutrients such as various types of amino acids, minerals, and phytochemicals such as steroids, flavonoids, and other bioactive ingredients (Fuentes‐Alventosa et al., 2009; Yi et al., 2019). Flavonoids are important components in asparagus, which may provide antiallergic, anti‐inflammatory, antiviral, and antioxidant activities to human body (Ferreyra et al., 2012; Franca et al., 2023; Li et al., 2023; Silva et al., 2023; Xu et al., 2023; Yang et al., 2023; Zhang et al., 2023). Rutin comprises the largest proportion of flavonoids in asparagus (Fuentes‐Alventosa et al., 2007). Another important component, steroidal saponins have been reported to affect cholesterol metabolism and protect the liver (Huang & Kong, 2006; Roman et al., 1995; Zhu et al., 2011). The most common steroidal saponins in asparagus is protodioscin (Huang & Kong, 2006; Liu et al., 2022). The physiological functions of asparagus (Jiménez‐Sánchez et al., 2021; Lee et al., 2014; Zhang et al., 2019), such as antitumor, antifatigue, hypoglycemic, hypolipidemic anti‐inflammatory, antioxidant, and protecting liver cells, mainly come from flavonoids and steroidal saponins. Besides, asparagus also has strong ecological values. Its strong salt‐alkali resistance property enables asparagus to grow normally in soil with less than 0.3% salt content (Chen et al., 2014; Gao et al., 2021; Zhang et al., 2020, 2022), and its sophisticated root system makes it effective in preventing soil erosion (Li et al., 2020).

As people's requirements for a healthy diet are gradually increasing, asparagus has a promising prospect as a nutritious and economical vegetable. The production of high‐quality asparagus is far from meeting the market demand (Li & Lin, 2022). The variety of asparagus is the most important factor that determines the yield and quality. Previous studies of some asparagus varieties mainly focused on agronomic traits, active component functions, and multiomics (Huang & Kong, 2006; Jiménez‐Sánchez et al., 2021; Lee et al., 2014; Liu et al., 2022; Yi et al., 2019; Zhang et al., 2019, 2022). However, it is still short of research on the active component comparison which may be essential for the screening of excellent varieties. In this study, seven chemical compositions, including dry matter, free amino acids, polyphenols, flavonoids, rutin, protodioscin, and the mineral selenium of 30 asparagus varieties in Shandong Province, China, were determined. The relationship between quality properties and varieties was investigated, and a comprehensive model for evaluating the quality of asparagus was established.

## 2 MATERIALS AND METHODS

### 2.1 Plant materials and reagents

The asparagus varieties were obtained from an asparagus planting base in Yuncheng County, Shandong, China. Their number, variety, and stem color are shown in Table 1. Samples were collected on April 27, 2023, and immediately placed in a portable cooler box and transported to the laboratory. The asparagus samples were cleaned with deionized water, crushed, mixed, and quartered after snap‐freezing in liquid nitrogen. A quarter of the samples were freeze‐dried at −70°C. The resulting lyophilizates were ground using a mixer and stored at −80°C until further analysis.

**TABLE 1:** Information about 30 asparagus spears.

No	Variety	Color	No	Variety	Color
1	WS‐1	Green	16	Jingke 1	Green
2	UC157	Green	17	Jingke 2	Green
3	Grande	Green	18	Jingke 3	Green
4	Walker noble	Green	19	Zhefeng 1	Green
5	Walker pioneer	Green	20	Fengdao 1	Green
6	Atlas	Green	21	Fengdao 2	Green
7	Appolo	Green	22	Hangyu 1	Green
8	Guelpin millennium	Green	23	Hangyu 6	Green
9	Purple passion	Purple	24	Zhefeng 801	Purple
10	Champion	Green	25	Zhefeng 806	Green
11	Shuofeng	Green	26	Zhefeng 811	Green
12	Jinglvlu 1	Green	27	Jinggang 701	Green
13	Jingzilu 2	Purple	28	Jinggang 111	Green
14	Jinglvlu 3	Green	29	Jinggang red	Purple
15	Jinglvlu 4	Green	30	T4	Green

Acetonitrile and methanol (high‐performance liquid chromatography [HPLC] grade) were purchased from Merck (Darmstadt, Germany). Deionized water was prepared by a Milli‐Q water purification system (Millipore, France). Formic acid was obtained from Rhawn (Shanghai, China). The Folin–Ciocalteu reagent was purchased from Sangon Biotech (Shanghai, China). The standard samples of gallic acid, leucine, protodioscin, and rutin were brought from Source Leaf Biotechnology Co., Ltd. (Shanghai, China). Other reagents used in this work were bought from Sinopharm Chemical Reagent Co. Ltd. (Shanghai, China).

### 2.2 Determination of the dry matter content, total phenols, total flavonoids, and free amino acids

The dry matter content of fresh asparagus was determined by heating 10 g of chopped samples at 105°C for 6 h (Liu et al., 2022). The total phenols were determined using the Folin–Ciocalteu method at 765 nm according to GB/T 8313 (National Standards, n.d.); the concentration was calculated from the calibration curve using gallic acid as the standard. The total flavonoids were analyzed according to Industry Standard – Agriculture, NY/T 1295‐2007 (n.d.), and the concentration was calculated from a calibration curve using rutin as the standard. The free amino acids were determined by the ninhydrin colorimetry method using leucine as the standard. Concentrations of total phenols and total flavonoids were expressed as mg/kg on a dry‐weight basis (DW). The concentration of free amino acids was expressed as g/100 g DW.

### 2.3 High‐performance liquid chromatography analysis of rutin and protodioscin

Rutin and protodioscin were determined by a modified HPLC‐UV method according to Yi et al. (2019). Briefly, the powdered sample (0.1 g) was extracted with 5 mL of 70% methanol at 30°C for 2 h. After filtration using a 0.22‐μm syringe filter (Woongki Science, Seoul, Korea), the filtrate was used for analysis. The sample was separated on a C18 column (250 mm × 4.6 mm, particle size 5 μm; Shimadzu, Kyoto, Japan) using a Prominence HPLC system (Shimadzu, Kyoto, Japan) equipped with a photo‐diode array detector. Solvent A was water (formic acid 0.1%) and solvent B was acetonitrile (formic acid 0.1%). The flow rate was 0.7 mL/min, the injection volume was 10 μL, and the column temperature was maintained at 40°C. The analytical wavelengths were 205 and 330 nm. Initially, the concentration of solvent B was 12%, which was then increased to 30% and 80% at 20 and 50 min, respectively. Concentrations of rutin and protodioscin were expressed as mg/kg DW.

### 2.4 Determination of mineral contents

The determination of the mineral contents was carried out using ICP emission spectroscopy (inductively coupled plasma). Each sample was digested in 3 mL of concentrated nitric acid (HNO_3_) and 3 mL of concentrated hydrochloric acid (HCl) on a digestion block at 95°C for 2 h; then made up to a final volume of 40 mL with deionized water. Each sample was digested in triplicate. High purity single element standards were used for quantitation. All results were expressed as mg/100 g DW.

### 2.5 Data processing and statistical analysis

All analyses were repeated three times, and the data were expressed as means and standard deviations. The radar map was made by Origin 2015. Experiment data were analyzed using a one‐way analysis of variance test in SPSS software (IBM Company) with the level of significance at 5%. The Pearson correlation analysis, principal component analysis (PCA), hierarchical cluster analysis (HCA), and comprehensive quality evaluation model were carried out by SPSS software. The orthogonal partial least‐squares discriminant analysis (OPLS‐DA) was performed by the statistics function in SIMCA software.

## 3 RESULTS AND DISCUSSION

### 3.1 The contents of seven chemical compositions in each asparagus variety

**FIGURE 1:**
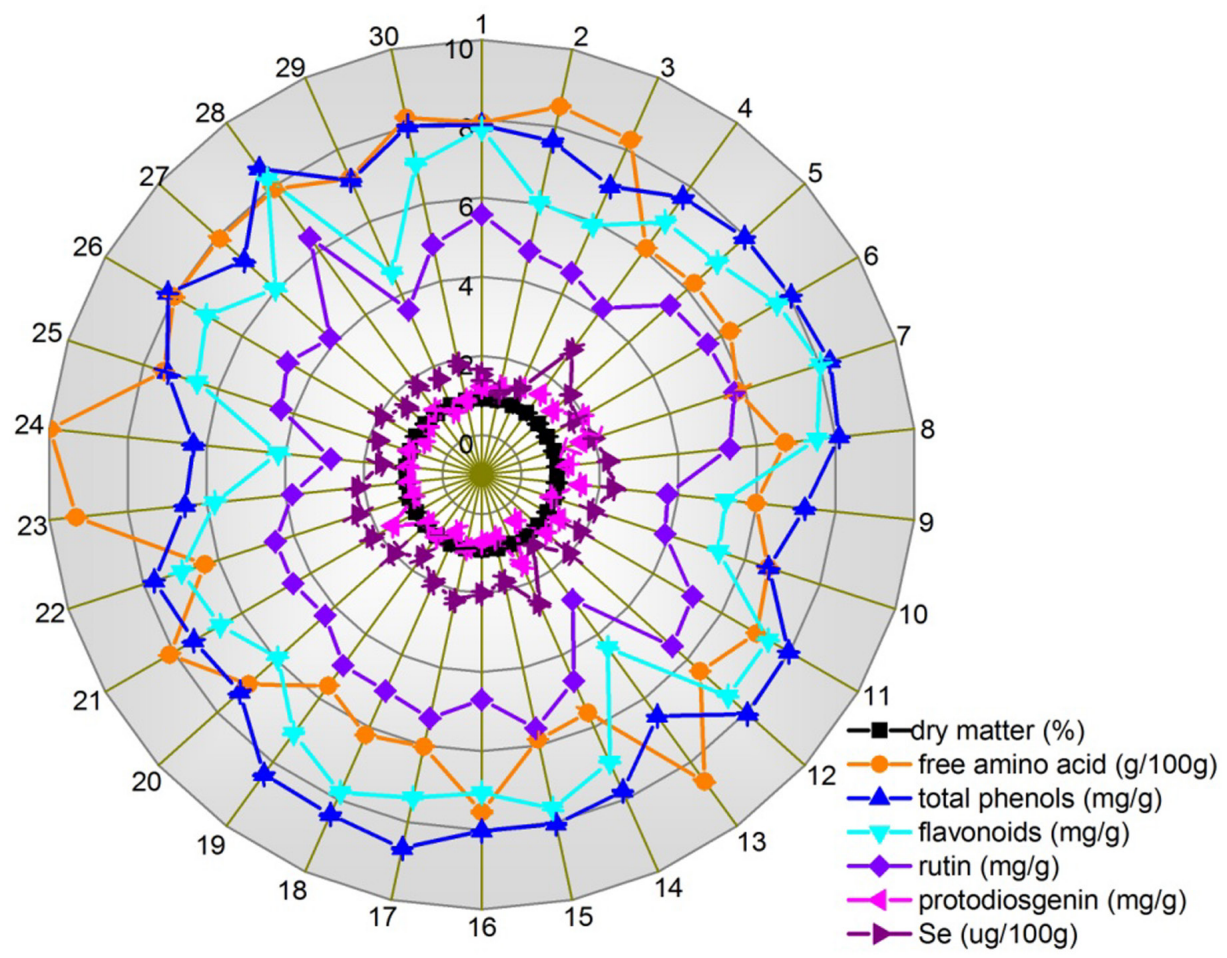
Chemical composition analysis radar map of asparagus samples. The varieties of each number are shown in Table 1.

By analyzing the content of dry matter, free amino acids, total phenols, flavonoids, rutin, protodioscin, and the mineral selenium of different asparagus varieties (Figure 1), we found that the top two varieties in amino acid content were Zhefeng 801 (9.98 g/100 g DW) and Hangyu 6 (9.32 g/100 g DW), while the lowest was in Jinglvlu 3 (5.60 g/100 g DW). The highest content of total phenols was in Jingke 2, followed by Jinggang 111, which had the highest content of flavonoids and rutin. The asparagus variety with the highest content of protodioscin was Atlas, 1.96 mg/g DW, which was 4.45 times as many as the lowest variety Jingzilu2. The highest content of the mineral selenium was in Walker noble, which was far more than the average value. Detailed values can be found in the Table S1.

Therefore, asparagus varieties with a low dry matter content, like Jingzilu 2, and Guelpin Millennium, will taste more hydrated. Asparagus varieties rich in saponins and selenium may have more antitumor effects (Kim et al., 2021; Peng et al., 2023), such as Atlas and Appolo, Walker noble, and Jinglvlu 3, which are the top two varieties in protodioscin and the mineral selenium, respectively. Jinggang 111, Appolo, Jingke 3, and Jingke 2 may have relatively stronger antioxidant and whitening effects, as their total phenols and flavonoid contents are higher (Ferreyra et al., 2012; Smeriglio et al., 2019).

### 3.2 Correlation analysis

Pearson correlation analysis was carried out on the quality indicators of asparagus, including dry matter, free amino acids, total phenols, flavonoids, rutin, protodioscin, and the mineral selenium, and results are shown in Table 2. There was little correlation (*r* < .5) between dry matter and the other six indicators (Table 2). Free amino acids were negatively correlated with total phenols, flavonoids, rutin, protodioscin, and the mineral selenium, respectively; however, it had the strongest correlation with total phenols (*r* = −.569, *p* < .01). In higher plants, most phenolic compounds come from the shikimic acid pathway (Lu & Chen, 2012). The aromatic amino acids–phenylalanine and methionine that are synthesized through the shikimic acid pathway are the first molecules for the biosynthesis of phenylpropanoids, while flavonoids are synthesized by phenylpropane biosynthetic pathway (Ferreyra et al., 2012; Lu & Chen, 2012). This revealed the negative correlation of free amino acids among total phenols, flavonoids, and rutin. There was a significant positive correlation among total phenols, flavonoids, and rutin, especially between flavonoids and rutin (*r* = .93, *p* < .01), as rutin comprises the largest proportion (55%–98%) of flavonoids in asparagus (Liu et al., 2022; Yi et al., 2019). The correlation coefficient between rutin and protodioscin was 0.345, which was consistent with the previous research results that genetic factors and light conditions affected the content of rutin and protodioscin in asparagus (Lee et al., 2010; Liu et al., 2022; Yi et al., 2019). The correlation between the mineral selenium and the other six indicators was also low (*r* < .5).

**TABLE 2:** Pearson correlation analysis.

Coefficient	Dry matter	Free amino acid	Total phenols	Flavonoids	Rutin	Protodioscin	Selenium
Dry matter	1	0.166	−0.225	−0.144	−0.083	0.004	−0.094
Free amino acid	–	1	−0.569**	−0.489**	−0.439**	−0.284*	−0.499**
Total phenols	–	–	1	0.890**	0.857**	0.170	0.226
Flavonoids	–	–	–	1	0.930**	0.279*	0.274*
Rutin	–	–	–	–	1	0.345**	0.077
Protodioscin	–	–	–	–	–	1	0.267*
Selenium	–	–	–	–	–	–	1

* *p* < .05.

** *p* < .01.

### 3.3 PCA and OPLS‐DA

The PCA method has been widely used to evaluate the quality of agricultural products such as kiwifruit, jujubes, foxtail millet, and jasmine tea (An et al., 2023; Peng et al., 2023; Singh et al., 2023; Zhao et al., 2023). By reducing the dimensionality of the data and eliminating overlapping information from numerous sources, the evaluation process was simplified, making it faster and more accurate than a single evaluation. At the same time, it avoided the correlation between traits that may affect the evaluation results.

**FIGURE 2:**
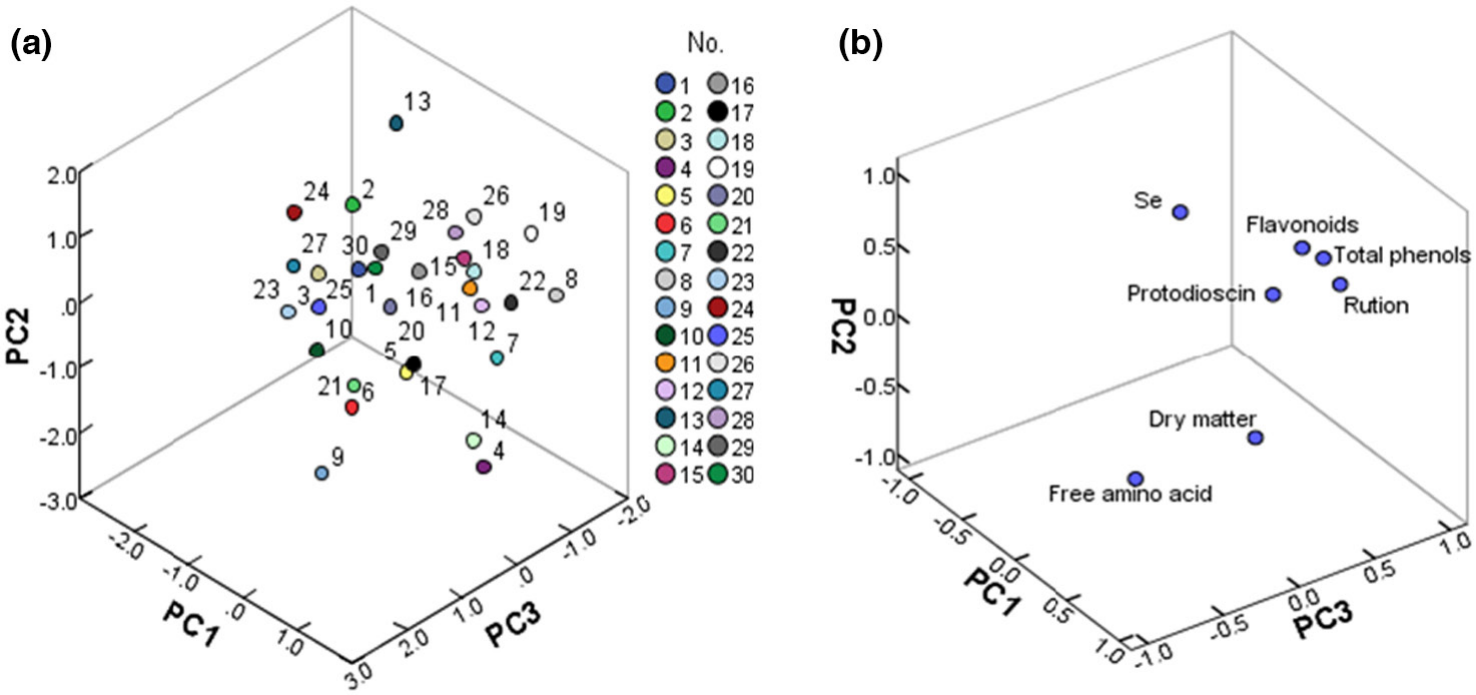
The score diagram (a) and loading diagram (b) of PCA. The varieties of each number are shown in Table 1. PCA, principal component analysis.

Considering the seven quality indicators comprehensively, the PCA was performed on the standardized data to explore the differences in the quality of different varieties of asparagus from an overall perspective. The score diagram of PCA is shown in Figure 2a, in which each dot represents an asparagus sample. The spatial distribution of these dots indicated the difference in the chemical compositions of each sample. It can be seen that 30 asparagus varieties were distributed in different regions, indicating that these chemical composition contents were significantly different among these samples. The loading diagram of PCA is shown in Figure 2b. Total phenols, flavonoids, and rutin had a high proportion in principal component 1, and selenium, free amino acid, and protodioscin comprised the largest proportion of principal component 2. Meanwhile, dry matter and protodioscin composed a majority of principal components 3. These two diagrams visually presented the quality and also revealed that different varieties or genotypes had important effects on the quality of the asparagus.

**FIGURE 3:**
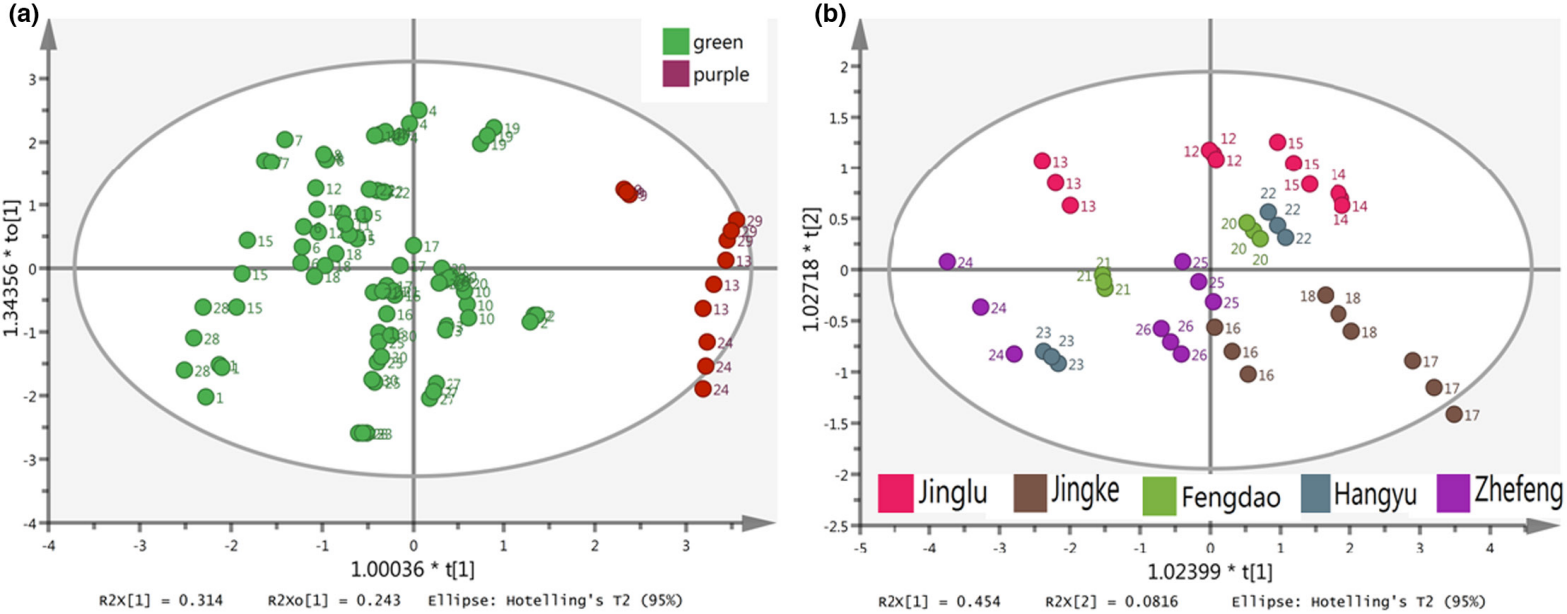
The score diagrams of OPLS‐DA: (a) was classed by stem color and (b) was classed by series. The varieties of each number are shown in Table 1.

OPLS‐DA is a supervised statistical method of discriminant analysis that differs from PCA (An et al., 2023; Lin et al., 2023). It can be seen that there were different distribution areas between asparagus samples with different stem colors (Figure 3a), indicating that stem color had a significant impact on the quality of asparagus. However, the relationship between series and quality was not significant, and the spatial distribution of different samples in the same series was also different (Figure 3b).

### 3.4 HCA

**FIGURE 4:**
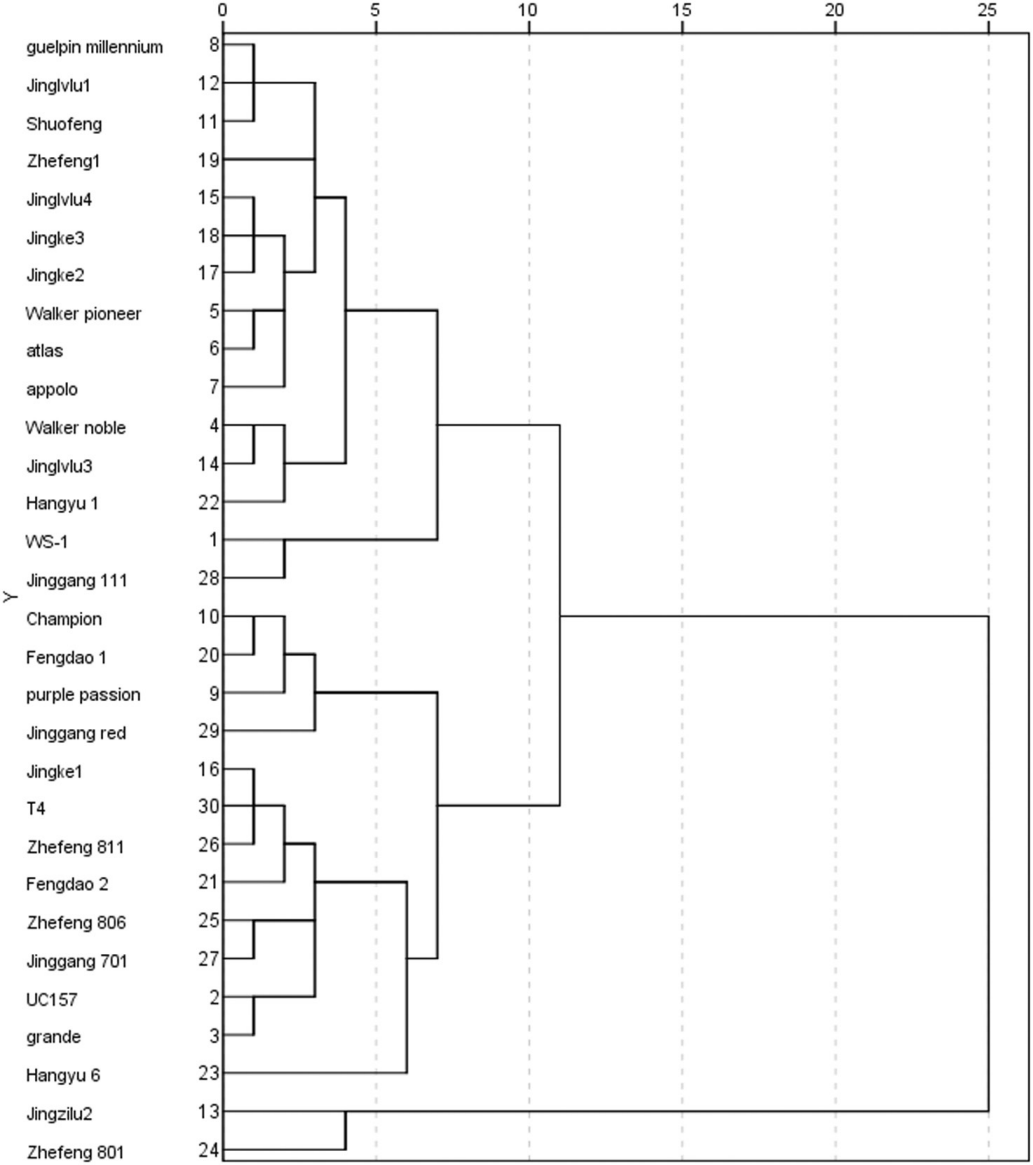
The tree diagram of HCA. The left numbers stand for asparagus samples, and the top numbers were distance markers. The cluster analysis was performed by using the intergroup linkage method and the measurement interval used a square Euclidean distance. HCA, hierarchical cluster analysis.

The HCA classifies the research objects according to their degree of affinity in quality indicators to analyze the similarity and difference between the quality of the samples (An et al., 2023). Therefore, HCA (Figure 4) was further conducted on the average contents of seven substances in 30 asparagus samples.

The 30 asparagus samples can be clustered into three groups when the distance was 10. Fifteen varieties, including numbers 1, 4, 5, 6, 7, 9, 11, 12, 14, 15, 17, 18, 19, 22, and 28, were grouped in one group; 13 varieties, including numbers 2, 3, 9, 10, 16, 20, 21, 23, 25, 26, 27, 29, 30, were grouped in one group; numbers 13 and 24 formed a third group. When the distance was 15, they were divided into two groups. The 15 varieties and the 13 varieties were combined into one group, indicating that the chemical composition contents of these samples were more similar.

### 3.5 Comprehensive quality evaluation model

To quantitatively evaluate the quality of these asparagus, a comprehensive evaluation model was established. Through PCA, seven quality indicators (dry matter, free amino acid, total phenol, flavonoids, rutin, protodioscin, and selenium) of 30 asparagus samples were transformed into three principal components. The principal components were extracted according to the total eigenvalue of the correlation coefficient matrix and the variance contribution rates of each principal component, as shown in Table 3. The cumulative variance contribution rate of these three principal components reached 81.87%, which meant these three principal components could represent 81.87% of the information of all detected quality indicators. Therefore, it was feasible to use these three principal components for the comprehensive evaluation of different asparagus varieties.

**TABLE 3:** Variance contribution rates of the principal components of nutritional quality.

Principal components	Total eigenvalue of correlation coefficient matrix	Variance contribution rates %	Cumulative variance contribution rates %
1	3.430	48.994	48.994
2	1.210	17.282	66.277
3	1.092	15.594	81.870

To further explain the relationship between the quality indicators of asparagus and the principal component factors, the functional expressions of the three principal components *F*1, *F*2, and *F*3 were obtained as follows, and *X*1, *X*2, *X*3, *X*4, *X*5, *X*6, and *X*7 represent the content of dry matter, free amino acid, total phenol, flavonoids, rutin, protodioscin, and selenium, respectively.

$$\begin{array}{c} F1=-0.12X1-0.388X2+0.494X3+0.503X4+0.481X5\\ \;+0.232X6+0.228X7. \end{array}$$

$$\begin{array}{c} F2=0.115X1+0.344X2-0.253X3-0.210X4-0.306X5\\ \;+0.427X6+0.696X7. \end{array}$$

$$\begin{array}{c} F3=0.818X1+0.228X2-0.064X3+0.099X4+0.230X5\\ \;+0.426X6-0.175X7. \end{array}$$

Based on the variance contribution rates of the principal components, the comprehensive evaluation model was built as *F* = 0.598 *F*1 + 0.211 *F*2 + 0.191 *F*3, and the scores of principal components and comprehensive scores are shown in Table 4. Atlas, Appolo, Jinggang 111, Jingke 2, and WS‐1 were the top five varieties of comprehensive score in turn, revealing that the comprehensive quality of these five asparagus varieties was relatively better.

**TABLE 4:** Scores of principal components and comprehensive scores.

No	*F*1	*F*2	*F*3	*F*	Ranking
1	1.044	−1.068	1.140	0.617	5
2	0.204	−1.578	0.383	−0.138	19
3	−0.376	−0.878	1.105	−0.199	22
4	−0.642	2.595	−0.602	0.0486	16
5	0.569	0.597	0.691	0.598	6
6	1.073	0.417	1.967	1.105	1
7	1.200	0.819	−0.003	0.890	2
8	0.613	0.701	−1.242	0.277	12
9	−1.305	1.757	1.233	−0.174	21
10	−1.348	0.389	0.279	−0.671	27
11	0.616	0.055	−0.522	0.280	11
12	0.769	0.313	−0.821	0.369	8
13	−1.807	−1.476	−2.155	−1.804	30
14	−0.153	2.052	−0.146	0.314	10
15	0.993	−0.407	−0.554	0.402	7
16	0.257	−0.350	−0.189	0.044	17
17	0.886	0.494	0.422	0.715	4
18	0.857	−0.126	−0.885	0.317	9
19	0.813	−0.184	−1.602	0.141	14
20	−0.643	0.121	0.084	−0.343	24
21	−0.366	0.663	1.066	0.125	15
22	−0.168	0.784	−1.491	−0.22	23
23	−1.385	−0.321	1.206	−0.666	26
24	−2.194	−1.114	−0.060	−1.559	29
25	−0.245	−0.493	1.067	−0.047	18
26	0.334	−0.606	−1.134	−0.145	20
27	−0.545	−1.022	0.969	−0.356	25
28	1.818	−1.053	0.016	0.868	3
29	−1.316	−0.319	−0.738	−0.995	28
30	0.447	−0.763	0.518	0.205	13

## 4 CONCLUSIONS

In this study, multiple quality indicators of 30 different asparagus varieties were transformed into a few comprehensive indexes covering the main information in order to analyze the performance of each quality indicator and comprehensively analyze the differences between different samples. The results showed that the constituents of different asparagus varieties were diverse. The top five varieties with relatively better comprehensive quality were selected as Atlas, Appolo, Jinggang 111, Jingke 2, and WS‐1. These varieties could be used as the first choice for consumption or cultivation. Jingzilu 2 and Guelpin millennium exhibit higher levels of moisture, while Walker noble and Jinglvlu3 exhibit higher levels of selenium. Additionally, Jinggang 111 and Appolo possessed high levels of flavonoids, while Atlas and Appolo were the most prominent two varieties in terms of protodioscin content. Consequently, consumers can opt for various asparagus varieties based on their needs or preferences, and processors can utilize various varieties to make deep‐processing products with distinctive characteristics.

Due to the rise in hypertension and hyperlipidemia, as well as a growing interest in a healthy diet, asparagus varieties that contain significant functional components will be highly regarded. Further investigations on the differences between genotypes and quality phenotypes are required to establish a theoretical foundation for asparagus breeding.

## AUTHOR CONTRIBUTIONS

**Rui Gao:** Conceptualization (lead); data curation (lead); funding acquisition (equal); investigation (lead); methodology (lead); software (equal); writing – original draft (lead); writing – review and editing (equal). **Guanghui Li:** Data curation (equal); resources (lead); supervision (equal); writing – review and editing (supporting). **Pingxiang Liu:** Formal analysis (equal); resources (equal); software (equal); validation (equal); writing – review and editing (supporting). **Lei Gao:** Project administration (equal); supervision (equal). **Jingxiu Bi:** Investigation (equal); methodology (equal); validation (equal). **Yuying Jiang:** Project administration (equal); supervision (equal). **Honglei Liu:** Writing – review and editing (lead). **Yutao Wang:** Conceptualization (equal); funding acquisition (equal); supervision (equal); writing – review and editing (supporting).

## FUNDING INFORMATION

This work was supported by the Agricultural Scientific and Technological Innovation Project of the Shandong Academy of Agricultural Sciences (nos. CXGC2022E05 and CXGC2022B04) and the Key R&D Plan of Shandong Province (no. LJNY202120).

## CONFLICT OF INTEREST STATEMENT

The authors declare that they have no known competing financial interests or personal relationships that could have appeared to influence the work reported in this paper.

## ETHICS STATEMENT

Not applicable.

## Supporting information


Table S1.

## Data Availability

The asparagus cultivation area and total production can be acquired from FAO statistics (Food and Agriculture Organization of the United Nations).
