# Supplementary material for: The quality evaluation of 30 Asparagus officinalis L. varieties
Source: Food Sci Nutr. 2024 Jan 23;12(4):2908–16. doi: 10.1002/fsn3.3971 (PMC11016430; doi:10.1002/fsn3.3971)
Supplement: Supplementary file 1 — Table S1. [file FSN3-12-2908-s001.docx]

Supplementary Material

**Table S1. The contents of dry matter, free amino acid, total phenols, flavonoids, rutin, protodioscin and mineral selenium in 30 asparagus.**

| **No.** | **variety** | **dry matter**  **(%)** | **free amino acid (g/100g)** | **total phenols ( mg/g)** | **Flavonoids**  **( mg/g)** | **Rutin**  **( mg/g)** | **protodioscin ( mg/g)** | **selenium (ug/100g)** |
| --- | --- | --- | --- | --- | --- | --- | --- | --- |
| 1 | WS-1 | 8.74±0.127 | 7.91±0.036 | 7.84±0.01 | 7.75±0.017 | 5.58±0.036 | 1.22±0.121 | 1.53±0.1 |
| 2 | UC157 | 8.2±0.035 | 8.51±0.015 | 7.63±0.046 | 6.04±0 | 4.78±0.03 | 1.25±0.021 | 1.12±0.035 |
| 3 | grande | 8.55±0.263 | 8.26±0.036 | 6.98±0.017 | 5.9±0 | 4.59±0.031 | 1.39±0.026 | 1.38±0.025 |
| 4 | Walker noble | 7.65±0.159 | 6.07±0.006 | 7.67±0.029 | 6.9±0.04 | 4.2±0.025 | 1.52±0.021 | 2.9±0.06 |
| 5 | Walker pioneer | 8.49±0.32 | 6.24±0 | 7.95±0 | 7.02±0 | 5.41±0.03 | 1.41±0.026 | 2.02±0.01 |
| 6 | atlas | 8.83±0.227 | 6.26±0 | 8.04±0.017 | 7.64±0 | 5.61±0.025 | 1.96±0.029 | 1.7±0.075 |
| 7 | appolo | 7.81±0.31 | 5.81±0 | 8.27±0.012 | 8.01±0.03 | 5.73±0.03 | 1.64±0.34 | 1.97±0.006 |
| 8 | guelpin millennium | 7.32±0.508 | 6.73±0.023 | 8.1±0.017 | 7.53±0.058 | 5.32±0.031 | 1.19±0.035 | 2.22±0.015 |
| 9 | purple passion | 8.95±0.015 | 5.98±0.006 | 7.22±0.025 | 5.21±0.023 | 3.74±0.031 | 1.5±0.032 | 2.35±0.04 |
| 10 | Champion | 8.7±0.431 | 6.62±0.01 | 6.64±0.029 | 5.31±0.035 | 3.88±0.03 | 0.85±0.021 | 1.97±0.085 |
| 11 | Shuofeng | 7.72±0.147 | 7.04±0.012 | 7.97±0.017 | 7.37±0.023 | 5.17±0.03 | 1.25±0.04 | 1.86±0.085 |
| 12 | Jinglvlu1 | 7.61±0.403 | 6.44±0.006 | 8.06±0 | 7.39±0.023 | 5.49±0.025 | 1.19±0.015 | 1.97±0.12 |
| 13 | Jingzilu2 | 7.27±0.502 | 8.6±0.026 | 6.56±0.017 | 4.43±0.023 | 2.92±0.03 | 0.44±0.025 | 1.22±0.006 |
| 14 | Jinglvlu3 | 7.97±0.181 | 5.6±0 | 7.77±0.017 | 6.96±0.023 | 4.72±0.03 | 1.49±0.072 | 2.58±0.035 |
| 15 | Jinglvlu4 | 8.32±0.395 | 5.85±0.006 | 8.04±0.017 | 7.64±0 | 5.57±0.026 | 0.64±0.035 | 1.77±0.08 |
| 16 | Jingke1 | 8.46±0.223 | 7.54±0.03 | 8.02±0.012 | 7.06±0.035 | 4.7±0.036 | 0.72±0.025 | 2.01±0.04 |
| 17 | Jingke2 | 8.88±0.326 | 6.04±0.006 | 8.67±0.017 | 7.36±0.035 | 5.31±0.031 | 0.91±0.072 | 2.26±0.05 |
| 18 | Jingke3 | 8.22±0.271 | 6.21±0.006 | 8.41±0.029 | 7.81±0.035 | 5±0.035 | 0.6±0.092 | 1.99±0.045 |
| 19 | Zhefeng1 | 7.45±0.123 | 5.61±0.006 | 8.38±0.029 | 7.1±0.023 | 4.97±0.03 | 0.95±0.021 | 1.55±0.08 |
| 20 | Fengdao 1 | 8.57±0.319 | 6.94±0.012 | 7.23±0.036 | 5.94±0.023 | 4.34±0.031 | 0.83±0.017 | 1.99±0.14 |
| 21 | Fengdao 2 | 8.44±0.069 | 8.11±0.015 | 7.42±0.029 | 6.64±0.074 | 4.51±0.035 | 1.6±0.02 | 2.2±0.05 |
| 22 | Hangyu 1 | 7.59±0.058 | 6.37±0.006 | 7.71±0.017 | 6.99±0.064 | 4.49±0.036 | 0.8±0.015 | 2.29±0.105 |
| 23 | Hangyu 6 | 9.09±0.393 | 9.32±0.017 | 6.55±0.017 | 5.8±0 | 3.81±0.025 | 0.84±0.032 | 2.15±0.045 |
| 24 | Zhefeng 801 | 8.21±0.338 | 9.98±0.026 | 6.34±0.029 | 4.18±0.04 | 2.84±0.035 | 0.86±0.035 | 1.55±0.095 |
| 25 | Zhefeng 806 | 9.1±0.287 | 7.46±0.012 | 7.36±0.075 | 6.57±0.017 | 4.34±0.036 | 0.93±0.026 | 1.75±0.01 |
| 26 | Zhefeng 811 | 7.82±0.064 | 7.99±0.015 | 8.17±0.012 | 7.03±0.051 | 4.67±0.026 | 0.68±0.021 | 1.93±0.06 |
| 27 | Jinggang 701 | 9.09±0.125 | 7.92±0.029 | 7.09±0.029 | 6.02±0.04 | 4.16±0.026 | 0.81±0.021 | 1.54±0.035 |
| 28 | Jinggang 111 | 8.14±0.387 | 7.93±0.026 | 8.56±0.029 | 8.22±0.023 | 6.4±0.038 | 1.02±0.017 | 1.76±0.05 |
| 29 | Jinggang red | 8.13±0.142 | 7.19±0.015 | 7.15±0 | 4.58±0.023 | 3.56±0.03 | 0.75±0.025 | 1.64±0.006 |
| 30 | T4 | 8.68±0.246 | 8.22±0.025 | 8.02±0.012 | 7.03±0.023 | 4.95±0.026 | 0.92±0.035 | 1.85±0.04 |
